# Utility of Periodontal exploration in patients with Fibromyalgia

**DOI:** 10.4317/jced.50691

**Published:** 2012-02-01

**Authors:** Rocío Santos-García, Benito Sánchez-Domínguez, Mario D. Cordero, José V. Rios-Santos, María R. Jaramillo-Santos, Mariano H. Climent, Pedro Bullon

**Affiliations:** 1 Departamento de periodontología, Facultad de Odontología, Universidad de Sevilla, Spain; 2 Centro Andaluz de Biología del Desarrollo (CABD), Universidad Pablo de Olavide-CSIC and Centro de Investigación Biomédica en Red de Enfermedades Raras (CIBERER), ISCIII, Sevilla, Spain; 3 Director del Master de Periodoncia e Implantología, Facultad de Odontología, Universidad de Sevilla, Spain

## Abstract

Objetive: Fibromyalgia (FM) is a chronic pain syndrome with unknown etiology, which affects predominantly women. Mitochondrial alteration could have a role in the pathophysilogical mechanisms of inflammatory conditions as FM and periodontitis. The aim of the present study was assay the relationship between both diseases and mitochondrial dysfunction. Patient and Methods: We study the presence of periodontitis in twelve patients diagnosed of FM and mitochondrial dysfunction described. The diagnosis of FM was established according to ACR criteria and clinical symptoms were evaluated using the Fibromyalgia Impact Questionnaire (FIQ) and Beck Depression Inventory (BDI). Results: Only one patients of twelve included and agreed to participate in the study were diagnosed with periodontitis. Conclusions: Pending studies with larger numbers of patients, we can conclude that mitochondrial dysfunction in FM is a itself event not related with periodontitis. Periodontitis could be considered a exclusion criterion in all studies about mitochondrial dysfunction in patients.

** Key words:**Peridontitis, fibromyalgia, mitocondrial dysfunction, oxidative stress.

## Introduction

Fibromyalgia (FM) is a common chronic pain syndrome accompanied by other symptoms such as depression, anxiety, fatigue or sleep disturbances (1). The prevalence of FM in Spain is 2.4% in the general population, being significantly more frequent in females (4,2%) than in males (0,2%) (2). However, the pathogenic mechanism of the disease is still unknown.

In recent years, new researches have shown evidence showing that mitochondrial dysfunction and oxidative stress may play an important role in the pathophysiology of FM (3,4). Oxidative stress, as an event characteristic of mitochondrial dysfunction is involved in other diseases such as: atherosclerosis, hypertension, insulin resistance, heart failure, and the aging process (5). Recently, it has also seen the presence of mitochondrial dysfunction and oxidative stress in patients with periodontitis (6). Periodontitis is a chronic infectious disease characterized by destructive inflammatory process affecting the tooth-supporting tissues resulting in loss of dentition. The cause is due to an ecological imbalance between the microbial biofilm on teeth and an impaired host inflammatory response. Oxidative stress is one of the main factors studied that could explain the pathophysiological mechanisms of inflammatory conditions that occur in the FM and periodontitis.

In order to demonstrate that mitochondrial dysfunction present in patients with FM is produced in the disease per se and not as a result of the presence of periodontitis, we propose in this paper the study of signs of periodontitis and the relationship with symptoms in a simple of patients with fibromyalgia.

## Patient and Methods

We studied a sample of 20 patients of the Sevillian Fibromyalgia Association (AFIBROSE) who met the criteria of the American Collage of Rheumatology (ACR). Two were males and 18 females, with mean age of (SD) 50,8±8,6 years, with mean duration of disease of 13,65±9,19 years and a mean of 14,9±3,1 tender points. The parameters of mitochondrial dysfunction in these patients were previously described in detail (4). Exclusion criteria were: rheumatic concomitant diseases, significant systemic diseases (cardiopulmonary, neurological, renal or feverish), patients with severe psychopathology or psychoactive substance dependence and chronic pain patients of different origin to the FM. The study was performed with the informed consent of all participants and the approval of the local ethical committee. Patients did not take any drugs or nutritional or vitamin supplements for a period of 15 days prior to sampling and reported a sedentary lifestyle.

In patients, we evaluated the impact of the disease by testing the Spanish version of Fibromyalgia Impact Questionnaire (FIQ), with an interval of 0-80, pain by visual analog scale (VAS), with a range of 0-10, and depression using the standard version of the Beck Depression Inventory (BDI), with an interval of 0-63 (0-9 no depression; 10-18 mild depression; 19-29 moderate depression; greater than 30 severe depression).

A baseline periodontal examination was performed, and a single examiner collected full medical and dental histories. A single trained dental examiner recorded periodontal data. The periodontal probing depth (PD) and the recession of the gingival margin (GM) relative to the cementoenamel junction at six sites per tooth were recorded. Clinical attachment level (CAL) was calculated by adding recession to PD. PD and CAL were recorded to the nearest highest millimeter by means of the North Carolina periodontal probe (HuFriedy, Chicago, IL, USA), 15 mm in length and 0.35 mm in diameter. According to the criteria established by Machtei et al. (7), the clinical entity of periodontitis is based on the presence of CAL ≥ 6mm in two or more teeth and one or more sites with PD ≥ 5mm. Patients were divided into two groups: one with periodontitis (n = 1) and the other without periodontitis (n = 19). We also collected plaque and bleeding indices. The bleeding index of Van der Velden (8) was measured at six sites per tooth, while the plaque index of Silness and Löe (9) was valued at four sites per tooth.

## Results

Patients showed high levels of pain determined by VAS, depression (BDI) and a high disease impact determined by the FIQ test (mean 7,2±2,2, 27±2,5 and 59,6±7,7, respectively) characteristic of the disease ( Table 1). With respect to periodontal disease, only 1 of 20 patients who met the inclusion criteria and agreed to participate in the study were diagnosed with periodontitis.  Table 2 summarizes the results of periodontal examination detailing of the parameters studied (GM, PD, CAL, dental plaque and bleeding on probing). As expected, the sample of patients with FM do not have periodontitis, with just one exception.

**Table 1:**
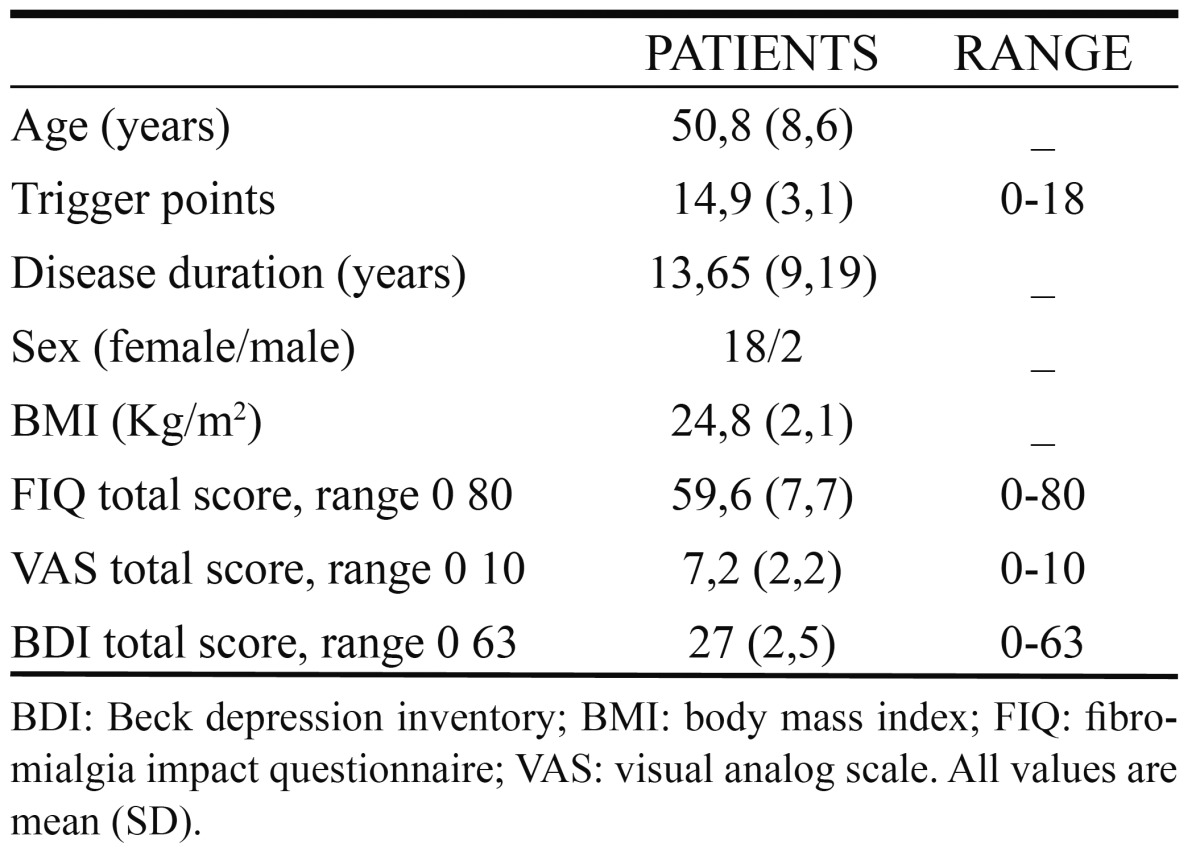
Clinical characteristics of patients.

**Table 2:**
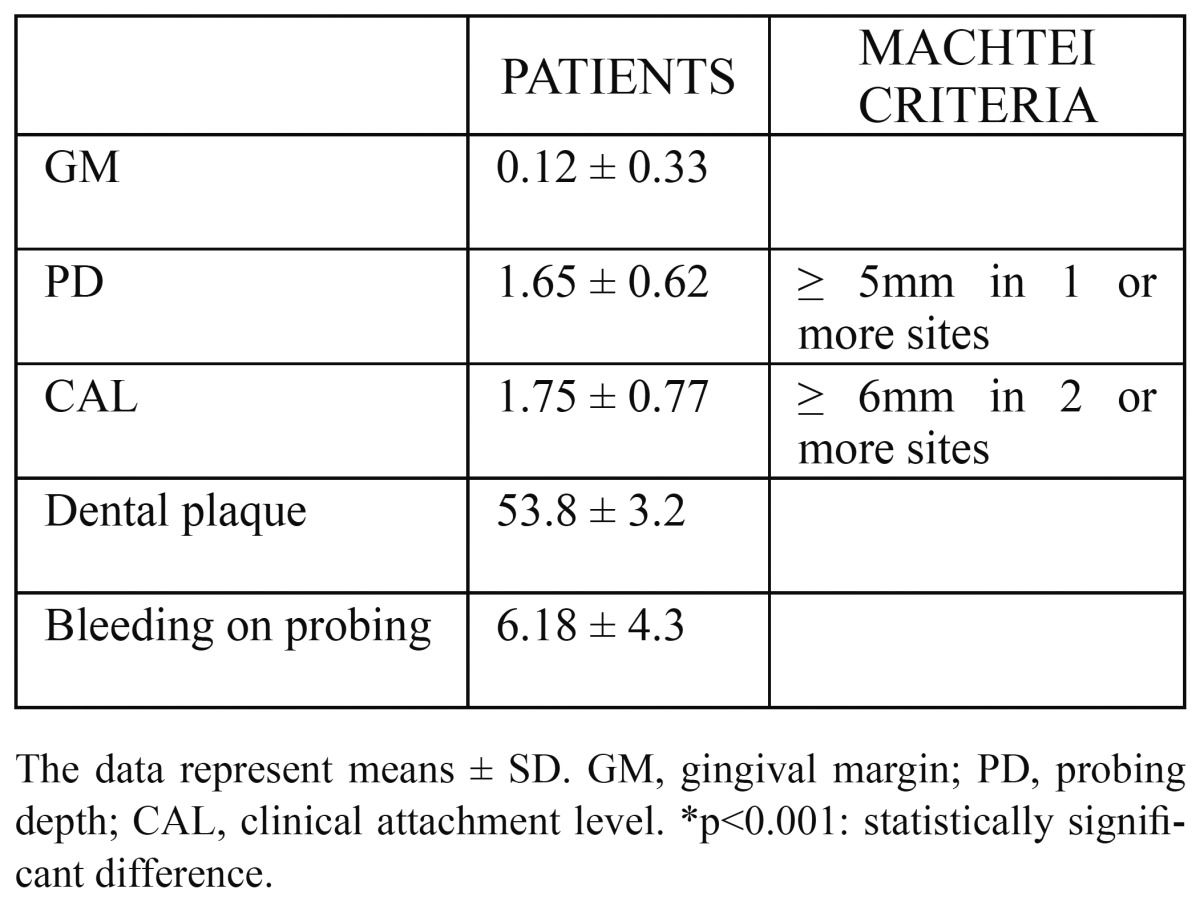
Results of periodontal examination.

## Discussion

In recent years, several studies have demonstrated the presence of markers of oxidative stress in FM, which raises the possibility of its involvement in the pathophysiology of the disease (3). Low levels of coenzyme Q have been detected (CoQ10) and an increased production of reactive oxygen species (ROS) in blood mononuclear cells of patients with FM, thus providing evidence of increased oxidative stress at the cellular level (10). On the other hand, oxidative stress and mitochondrial dysfunction are key factors that explain some of the pathophysiological mechanisms associated with inflammatory conditions such as periodontitis, being lipid peroxidation one of its better known effects. In fact, there has been observed an increased lipid peroxidation in periodontitis (11), knowing that it indirectly reflects intracellular ROS production. Thus, it has also seen the presence of mitochondrial dysfunction in patients with periodontitis (6).

After excluding other diseases in which it is well recognized the presence of mitochondrial dysfunction, in this article we propose the study of periodontal disease as another possible exclusion criteria in future studies that assess the presence of mitochondrial dysfunction and relate to the FM. To do this in this study we took into account several variables to determine whether mitochondrial dysfunction in patients with FM is due to the disease itself or whether it could be due to the presence of periodontitis. In our sample, we obtained as result the absence of periodontal disease, this would confirm that mitochondrial disfunction present in patients with FM is typical of the disease and does not occur as a result of periodontitis. It is noteworthy that the patients included in the sample have high rates of dental plaque while bleeding on probing has very low values, this demostrates the low susceptibility of the sample to both bleeding and thus periodontal disease.

In conclusion, following the obtained results and waiting for statistically superior studies in the sample, we can say that mitochondrial dysfunction found in a sample of patients with fibromyalgia is characteristic of the disease and does not occur as a result of the presence of periodontal disease.

On the other hand, another factor to consider in future research is that the presence of periodontitis should be considered as a exclusion criteria for any study whose objective the presence of mitochondrial dysfunction.

## References

[1] Wolfe F, Russell IJ, Vipraio G, Ross K, Anderson J (1997). Serotonin levels, pain threshold and fibromyalgia symptoms in the general population. J Rheumatol.

[2] Izquierdo Álvarez S, Bancalero Flores JL, García Pérez MC, Serrano Ostariz E, Alegre de Miguel C, Bocos Terraz JP (2009). Evaluación de la cortisoluria en mujeres diagnosticadas de fibromialgia. Med Clin (Barc).

[3] Cordero MD, de Miguel M, Moreno-Fernández AM (2010). La disfunción mitocondrial en la fibromialgia y su implicación en la patogénesis de la enfermedad. Med Clin (Barc).

[4] Cordero MD, De Miguel M, Moreno Fernández AM, Carmona López IM, Garrido Maraver J, Cotán D (2010). Mitochondrial dysfunction and mitophagy activation in blood mononuclear cells of fibromyalgia patients: Implication in the pathogenesis of the disease. Arthritis Research & Therapy.

[5] Halliwell B (1991). Reactive oxygen species in living systems: source, biochemistry, and role in human disease. Am J Med.

[6] Bullón P, Cordero MD, Quiles JL, Morillo JM, Ramirez-Tortosa MC, Battino M (2011). Mitochondrial dysfunction promoted by Porphyromonas gingivalis lipopolysaccharide as a possible link between cardiovascular disease and periodontitis. Free Radic Biol Med.

[7] Machtei EE, Christersson LA, Grossa SG, Dunford R, Zambon JJ, Genco RJ (1992). Clinical criteria for the definition of “established periodontitis”. J Periodontol.

[8] Van der Velden U, Abbas F, Van Steenbergen TJ, De Zoete OJ, Hesse M, De Ruyter C (1989). Prevalence of periodontal breakdown in adolescents and presence of Actinobacillus actinomycetemcomitans in subjects with attachment loss. J Periodontol.

[9] Silness J, Loe H (1964). Periodontal diseases in pregnancy. II Correlation between oral hygiene and periodontal condition. Acta Odontol. Scand.

[10] Cordero MD, Moreno-Fernández AM, deMiguel M, Bonal P, Campa F, Jiménez-Jiménez LM (2009). Coenzyme Q10 distribution in blood is altered in patients with fibromyalgia. Clin Biochem.

[11] Pischon T, Hu FB, Rexrode KM, Girman CJ, Manson JE, Rimm EB (2008). Inflammation, the metabolic síndrome, and risk of coronary heart disease in women and men. Atherosclerosis.

